# Flexor Tenosynovitis as the Sole Initial Presentation of Anti-synthetase Syndrome: A Case Report

**DOI:** 10.7759/cureus.78133

**Published:** 2025-01-28

**Authors:** Taylor Kann, Richa Purohit, Mariana Aziz, Marie Rivera-Zengotita, Maria Farooq

**Affiliations:** 1 Rheumatology, University of Central Florida College of Medicine, Orlando, USA; 2 Rheumatology, University of Central Florida HCA Healthcare Graduate Medical Education (GME), Orlando, USA; 3 Internal Medicine, Orlando Internal Medicine, Orlando, USA; 4 Internal Medicine, University of Central Florida College of Medicine, Orlando, USA; 5 Internal Medicine, University of Central Florida HCA Healthcare Graduate Medical Education (GME), Orlando, USA; 6 Pathology, University of Florida, Gainesville, USA

**Keywords:** anti-jo-1 antibodies, anti-synthetase (as) syndrome, flexor tenosynovitis, immune-mediated inflammatory myopathy, mechanic’s hands

## Abstract

Anti-synthetase syndrome (ASS) is a rare autoimmune disorder characterized by interstitial lung disease (ILD), myositis, and arthritis, primarily associated with antibodies, such as anti-Jo-1, that target the t-ribonucleic acid (tRNA) synthetase enzymes. This case report describes a 45-year-old man who presented with isolated flexor tenosynovitis and bilateral hand pain, later diagnosed with anti-Jo-1 positive ASS. Initially, the patient’s symptoms were attributed to a trivial injury, but subsequent imaging and laboratory evaluations revealed tenosynovitis without signs of inflammatory arthritis. Positive anti-Jo-1 antibodies and later elevated creatine kinase (CK) levels indicated the development of myositis, supported by magnetic resonance imaging (MRI) findings and muscle biopsies showing mild inflammatory features. The patient was treated with prednisone and mycophenolate mofetil, resulting in significant clinical improvement and resolution of symptoms.

This case highlights an atypical presentation of ASS, with isolated tenosynovitis as an initial symptom, which is not commonly documented in the literature. The findings underscore the importance of considering ASS in patients with unexplained inflammatory symptoms and highlight the role of comprehensive clinical evaluation and antibody testing in guiding diagnosis. By recognizing the variable clinical manifestations of ASS, clinicians can facilitate earlier diagnosis and prompt treatment, improving patient outcomes.

## Introduction

Anti-synthetase syndrome (ASS) is a chronic autoimmune disorder that represents a subtype of idiopathic inflammatory myopathies and is associated with autoantibodies that target the t-ribonucleic acid (tRNA) synthetase enzymes, with anti-Jo-1 being the most common [1,2]. Clinical manifestations of ASS typically include inflammatory myositis, polyarthritis, interstitial lung disease (ILD), Raynaud's phenomenon, and mechanic's hands, although the clinical picture may vary depending on the specific autoantibody profile [2,3]. According to Solomon et al., the diagnostic criteria require a positive anti-aminoacyl tRNA synthetase antibody plus two major or one major and two minor criteria. Major criteria include ILD, polymyositis, or dermatomyositis, while minor criteria encompass arthritis, Raynaud's phenomenon, and mechanic's hands [4].

Inflammatory arthritis is a recognized feature of ASS and can sometimes be misdiagnosed as rheumatoid arthritis [5]. Flexor tenosynovitis, although rarely described, can be an initial manifestation of ASS and is not widely reported in medical literature. The treatment of ASS generally includes high-dose glucocorticoids and disease-modifying antirheumatic drugs (DMARDs) such as methotrexate and mycophenolate mofetil, with therapy tailored to the severity of the disease and specific organ involvement [6].

This report presents the case of a 45-year-old man with isolated flexor tenosynovitis, later identified as Jo-1 antibody-positive ASS, providing new insights into the clinical spectrum of ASS. Flexor tenosynovitis as an isolated early presentation expands the understanding of this syndrome’s variability, highlighting the need for early consideration of ASS in similar cases.

## Case presentation

A 45-year-old Caucasian male with no significant past medical history was referred to the rheumatology clinic for bilateral hand pain and a positive antinuclear antibody (ANA) screening test. The patient reported that the pain began in his right hand after a trivial injury; however, it progressed to both hands over the next few months. On physical examination, the patient exhibited tenderness over the palmar surface of the right third digit, raising concerns about flexor tenosynovitis, commonly known as "trigger finger." He received a local triamcinolone injection, which provided only temporary relief of symptoms. This prompted further evaluation with magnetic resonance imaging (MRI) of the right hand, which was negative for any intercarpal, metacarpal, or interphalangeal joint synovitis or erosive changes suggestive of inflammatory arthropathy. The MRI revealed only a small amount of fluid in the flexor tendon sheath distal to the carpal tunnel, consistent with tenosynovitis.

Meanwhile, his lab workup revealed a complete blood count (CBC) and comprehensive metabolic panel (CMP) within normal limits. ANA was positive with a titer of 1:80 and a speckled pattern, which, while not specific, is commonly observed in various autoimmune conditions, including anti-synthetase syndrome (ASS). The positive ANA provided additional context supporting an autoimmune etiology in conjunction with other findings. Additional testing was also positive for anti-Jo-1 antibody >8 (Reference: 0.0 to 0.99 U/L). The remaining immunologic labs, including anti-double-stranded antibodies (DS-DNA) for lupus, anti-Smith antibody (SM/RNP), anti-Sjögren’s antibodies (SS-A/SS-B), anti-scleroderma antibodies (Scl-70), hepatitis panel, HLA-B27 antigen, and Lyme disease antibodies (IgG and IgM), were negative, and the creatine kinase (CK) levels were within normal limits at 195 U/L (Reference: 22 to 198 U/L). The patient was prescribed a short course of prednisone, which resulted in an initial improvement of his symptoms. However, the symptoms recurred after tapering the steroids. A presumptive diagnosis of undifferentiated inflammatory arthritis was made, and he was started on hydroxychloroquine 200 mg twice daily as a steroid-sparing agent.

Two months after initiating hydroxychloroquine, the patient reported weakness in his proximal upper and lower extremities, along with associated cracking of the skin around his fingers, consistent with the mechanic's hands. He also noted occasional shortness of breath. Laboratory evaluation at this time revealed significantly elevated CK levels (4500 U/L), aldolase (94.2 U/L; Reference: 1.2-7.6 U/L), lactate dehydrogenase (LDH) (663 IU/L; Reference: 105-333 IU/L), aspartate transaminase (AST) (223 U/L; Reference: <33 U/L), and alanine transaminase (ALT) (271 U/L; Reference: <48 U/L). These combined clinical and biochemical findings prompted the treating physician to investigate ASS as the underlying condition. The remainder of the myositis panel was negative (Table 1).

**Table 1:** Laboratory findings with reference ranges This table summarizes the key laboratory findings from the patient, including test results and their respective reference ranges.

Test	Result	Reference Range
ANA Titer	1:80 (Speckled)	Negative: <1:40
Anti-Jo-1 Antibody	>8.0 U/L	0.0–0.99 U/L
Creatine Kinase (CK)	4500 U/L	22–198 U/L
Aldolase	94.2 U/L	1.2–7.6 U/L
Lactate Dehydrogenase (LDH)	663 IU/L	105–333 IU/L
Aspartate Transaminase (AST)	223 U/L	<33 U/L
Alanine Transaminase (ALT)	271 U/L	<48 U/L
Anti-dsDNA Antibody	Negative	Negative
Anti-Sm/RNP Antibody	Negative	Negative
Anti-SSA/SSB Antibodies	Negative	Negative
Anti-Scl-70 Antibody	Negative	Negative
HLA-B27 Antigen	Negative	Negative
Lyme Disease Antibodies (IgG & IgM)	Negative	Negative

**Figure 1:**
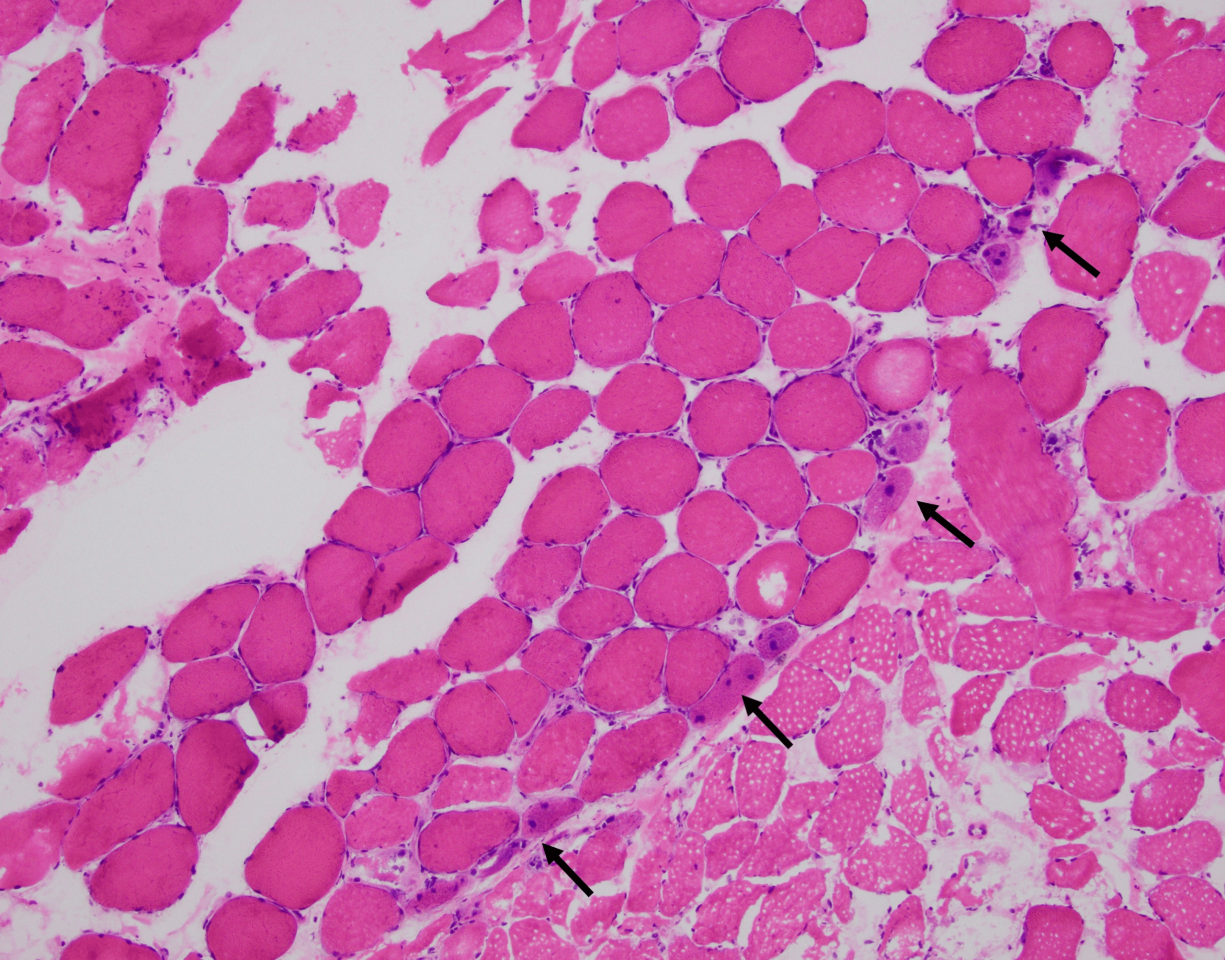
Histopathological features of myopathy in anti-synthetase syndrome Snap-frozen, hematoxylin and eosin (H&E) stained sections of skeletal muscle show mild myopathic features including necrotic and degenerating myofibers localized to the perifascicular region (arrows).

**Figure 2:**
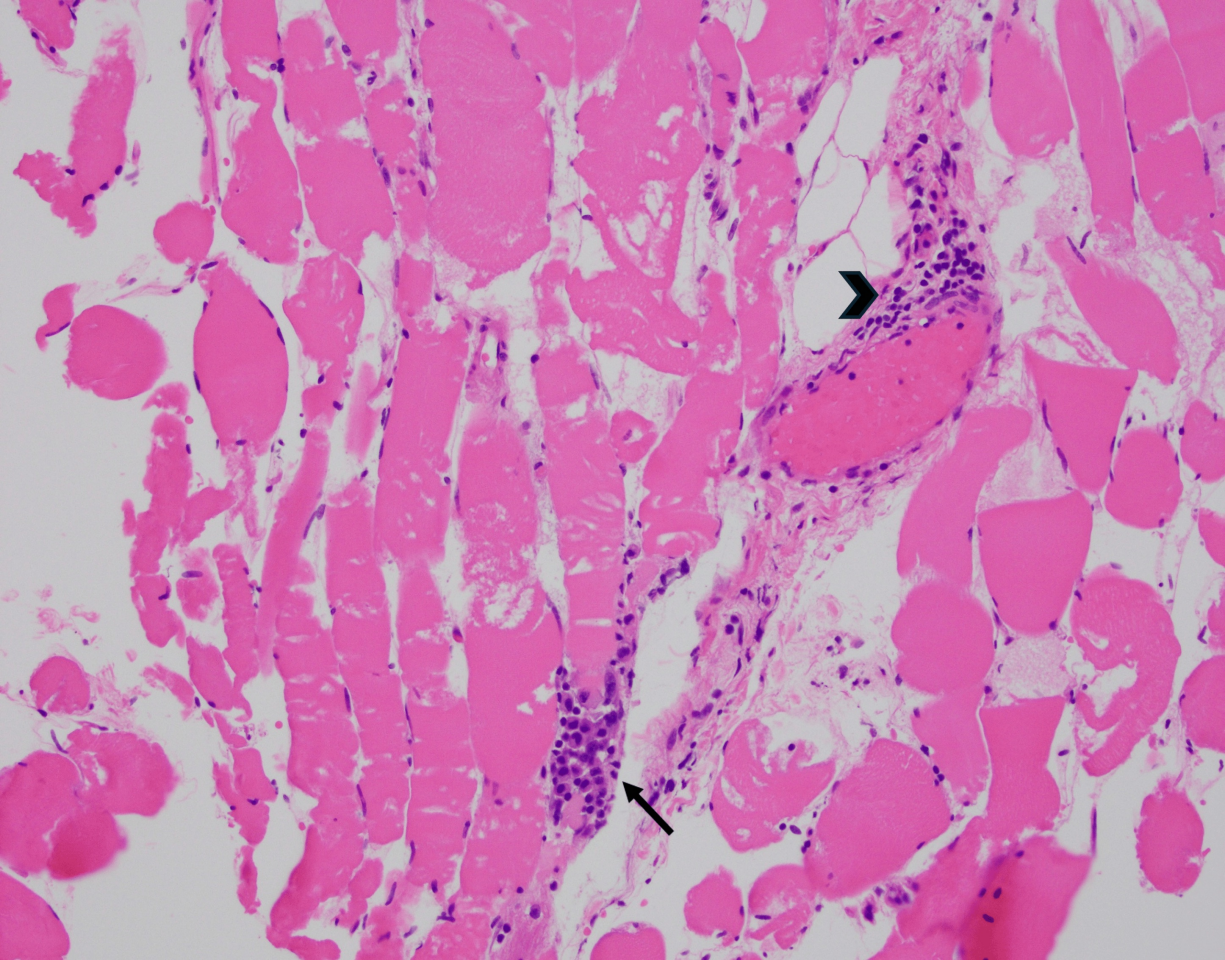
Focal Inflammation in anti-synthetase syndrome-associated myositis Paraffin-embedded hematoxylin and eosin (H&E) stained section of skeletal muscle reveals focal endomysial (arrow) and perivascular (arrowhead) inflammation by T-lymphocytes and macrophages.

A bilateral MRI of the thighs was ordered to further investigate for myositis, and it showed symmetric edema of the hip muscles, more pronounced in the sartorius and rectus femoris, suggesting inflammatory myopathy such as dermatomyositis and polymyositis. This prompted muscle biopsies of the right and left quadriceps femoris, which showed mild myopathic features, including scattered necrotic and regenerating myofibers which focally appeared to cluster in the perifascicular region (Figure 1). In addition, focal endomysial and perivascular inflammation by T-lymphocytes and macrophages was identified (Figure 2). These findings are consistent with a histopathologic diagnosis of anti-synthetase syndrome-associated myositis. No rimmed vacuoles were seen to suggest the inclusion of body myositis, and enzyme histochemistry (EHC) did not suggest mitochondrial or glycogen abnormalities.

The patient was restarted on prednisone at 60 mg daily, which was tapered off over nine months. Hydroxychloroquine was discontinued, and mycophenolate mofetil was initiated at 500 mg daily, with the dose gradually increased to 2 grams daily. A computed tomography (CT) scan of the chest, abdomen, and pelvis was performed to screen for potential underlying malignancy, given the association between inflammatory myopathies and cancer. CT chest showed minimal linear subpleural densities in the dependent lower lobes representing minimal scarring or atelectasis. There was no evidence of ILD found on the patient’s chest CT scan. The scan also did not reveal any other abnormalities, ruling out malignancy as a contributing factor. Additionally, pulmonary function testing and chest imaging were conducted for ILD.

The patient responded well to treatment, with significant improvement in muscle strength and complete resolution of tenosynovitis and mechanic’s hands, which are common extra-muscular manifestations of myositis. A carefully monitored taper of prednisone was initiated, lasting approximately 14 months. During this time, the patient remained stable with no recurrence of symptoms, suggesting successful disease control.

## Discussion

Anti-synthetase syndrome (ASS) is a rare autoimmune condition characterized by interstitial lung disease (ILD), myositis, and arthritis, with approximately 90% of patients exhibiting these features [7]. Tenosynovitis as the initial manifestation of anti-synthetase syndrome is rarely documented in the literature. While musculoskeletal symptoms, including arthritis and myositis, are common in anti-synthetase syndrome, case reports highlight significant variability in presentations, such as ILD and mechanic's hands. One such study evaluated eight ASS patients, five with known inflammatory arthritis, and found synovial hypertrophy in all, with effusions in 71% of joints. Notably, tenosynovitis of the finger flexors or wrist extensors was identified in seven patients [8]. In our case, the patient presented with isolated flexor tenosynovitis, which later progressed to myositis and other features consistent with ASS, highlighting the need for clinicians to consider this rare association in similar cases.

The presence of anti-Jo-1 antibodies, an anti-histidyl-tRNA synthetase, is a hallmark of ASS [9]. These antibodies are detected in 15% to 25% of polymyositis patients and up to 70% of those with myositis who also exhibit ILD [10]. In our case, the patient tested positive for anti-Jo-1 antibodies, highlighting a critical serological finding that supports the diagnosis of ASS.

While the initial symptoms included bilateral hand pain consistent with tenosynovitis, further evaluations revealed elevated creatine kinase (CK) levels, suggesting skeletal muscle involvement, a key indicator for inflammatory myopathy [11]. The extent of CK elevation, along with significant increases in aldolase, LDH, and liver enzymes, exceeded levels typically observed in steroid-induced myopathy, which is usually associated with only mild CK elevation. Furthermore, the clinical finding of mechanic's hands, a hallmark of anti-synthetase syndrome (ASS), strengthened the suspicion of an inflammatory myopathy. The previously identified positive anti-Jo-1 antibody and positive histologic findings in skeletal muscle biopsy further corroborated the diagnosis of inflammatory myopathy.

ILD represents the most significant pulmonary complication associated with ASS, occurring in up to 86% of patients and contributing substantially to morbidity and mortality [7]. In many cases, ILD may precede the onset of myositis or arthritis, but it can also manifest concurrently or even develop later in the disease course, underscoring the heterogeneity of this condition [12]. The natural progression of ASS varies, with some patients experiencing rapid ILD progression and others demonstrating a more indolent course. Although initial pulmonary assessments in our patient revealed no signs of ILD, it is crucial to maintain vigilance, as respiratory symptoms could emerge over time. This highlights the importance of regular lung function monitoring and periodic imaging as integral components of ASS management. Comparing the patient’s trajectory to the typical course of ASS, the absence of ILD at the time of diagnosis represents an atypical but not unprecedented presentation, warranting long-term follow-up to preemptively address potential pulmonary complications.

While Raynaud's occurs in approximately 40% of cases, not all patients will exhibit this symptom [13]. The absence of Raynaud's phenomenon in our case underscores the variability in clinical presentations associated with ASS. Similarly, the varied presentation of arthritis in ASS emphasizes the need for thorough evaluation and consideration of the syndrome in patients presenting with atypical inflammatory arthritis symptoms.

In terms of management, the treatment of ASS often involves a combination of glucocorticoids and immunosuppressive agents, with mycophenolate mofetil being a common choice for maintenance therapy [4,6]. In this case, the patient's significant improvement with prednisone and the gradual introduction of mycophenolate mofetil align with established therapeutic strategies for managing ASS.

This case report highlights an atypical presentation of anti-synthetase syndrome (ASS), emphasizing the importance of early recognition of subtle clinical signs, such as mechanic's hands, and the role of antibody testing in diagnosis. While it adds to the limited literature on isolated flexor tenosynovitis as an initial manifestation, the single-patient nature limits generalizability. The absence of long-term pulmonary monitoring and photographic documentation are notable limitations. Despite this, the case underscores key diagnostic and management principles for this rare condition.

## Conclusions

This case highlights the importance of recognizing atypical presentations of anti-synthetase syndrome (ASS), such as isolated flexor tenosynovitis, to enable timely diagnosis and treatment. Early intervention with glucocorticoids and mycophenolate mofetil resulted in significant clinical improvement, underscoring the efficacy of established therapies. Ongoing vigilance for complications like interstitial lung disease, even in asymptomatic patients, remains crucial to improving outcomes in this rare autoimmune condition.
